# The D2.*mdx* mouse as a preclinical model of the skeletal muscle pathology associated with Duchenne muscular dystrophy

**DOI:** 10.1038/s41598-020-70987-y

**Published:** 2020-08-21

**Authors:** David W. Hammers, Cora C. Hart, Michael K. Matheny, Lillian A. Wright, Megan Armellini, Elisabeth R. Barton, H. Lee Sweeney

**Affiliations:** 1 Department of Pharmacology and Therapeutics, University of Florida College of Medicine, 1200 Newell Dr., ARB R5-216, Gainesville, FL 32610-0267 USA; 2 Myology Institute, University of Florida College of Medicine, Gainesville, FL USA; 3 Department of Applied Physiology and Kinesiology, University of Florida, Gainesville, FL USA

**Keywords:** Neuromuscular disease, Molecular medicine, Musculoskeletal system

## Abstract

Duchenne muscular dystrophy (DMD) is an X-linked, lethal muscle degenerative disease caused by loss of dystrophin protein. DMD has no cure and few treatment options. Preclinical efforts to identify potential DMD therapeutics have been hampered by lack of a small animal model that recapitulates key features of the human disease. While the dystrophin-deficient *mdx* mouse on the C57BL/10 genetic background (B10.*mdx*) is mildly affected, a more severe muscle disease is observed when the *mdx* mutation is crossed onto the DBA/2J genetic background (D2.*mdx*). In this study, the functional and histological progression of the D2.*mdx* skeletal muscle pathology was evaluated to determine the distinguishing features of disease. Data herein details the muscular weakness and wasting exhibited by D2.*mdx* skeletal muscle, as well as severe histopathological features, which include the rapid progression of fibrosis and calcifications in the diaphragm and progressive fibrosis accumulation in limb muscles. Furthermore, a timeline of D2.*mdx* progression is provided that details distinct stages of disease progression. These data support the D2.*mdx* as a superior small animal model for DMD, as compared to the B10.*mdx* model. The insights provided in this report should facilitate the design of preclinical evaluations for potential DMD therapeutics.

## Introduction

Effective preclinical evaluation of potential therapeutics for human diseases requires thorough understanding of the disease phenotype exhibited by animal models utilized during such studies. Duchenne muscular dystrophy (DMD) is a fatal X-linked pediatric muscle disease with no cure and limited treatment options for affected patients. DMD is caused by mutations in the *DMD* gene resulting in complete loss of the gene’s protein product, dystrophin^1^, a molecule that provides stabilization of the sarcolemma during muscular contraction by linking the muscle cytoskeleton and extracellular matrix^2,3^. Preclinical work to study DMD has largely utilized the *mdx* mouse harboring a nonsense mutation in exon 23 of *Dmd*, which has been primarily maintained on the C57BL/10 genetic background (referred to as B10.*mdx*). This genetic homolog of DMD, however, exhibits a markedly mild disease phenotype compared to the human disease, limiting its potential to accurately model possible DMD therapeutics.

Recent efforts to identify murine models of DMD that more accurately recapitulate the severity of the human condition than the B10.*mdx* mouse have led to the discovery that crossing the *mdx* mutation onto the DBA/2J genetic background (referred to D2.*mdx*) results in a more severe disease phenotype. This model exhibits greater muscle damage, impaired muscle regeneration, muscle wasting, and exacerbated progression of intramuscular fibrosis than age-matched B10.*mdx* mice^4–7^. The heightened severity of the D2.*mdx* mouse has been linked to a hyper-fibrotic polymorphism in latent TGFβ binding protein (LTBP) 4^8^, which has also been identified as a genetic modifier affecting disease progression within DMD patient populations^9^. For these reasons, the D2.*mdx* mouse is emerging as a more relevant small animal model for translational DMD research^10–12^. In the current report, we provide a detailed examination of the functional and histological progression of skeletal muscle pathology exhibited by the D2.*mdx* mouse. These data highlight the different stages of skeletal muscle disease progression, which may have important implications for the proper selection, timing, and interpretation of preclinical testing of potential DMD therapeutics.

## Results

### Functional comparison of B10.mdx and D2.mdx muscle

**Figure 1:**
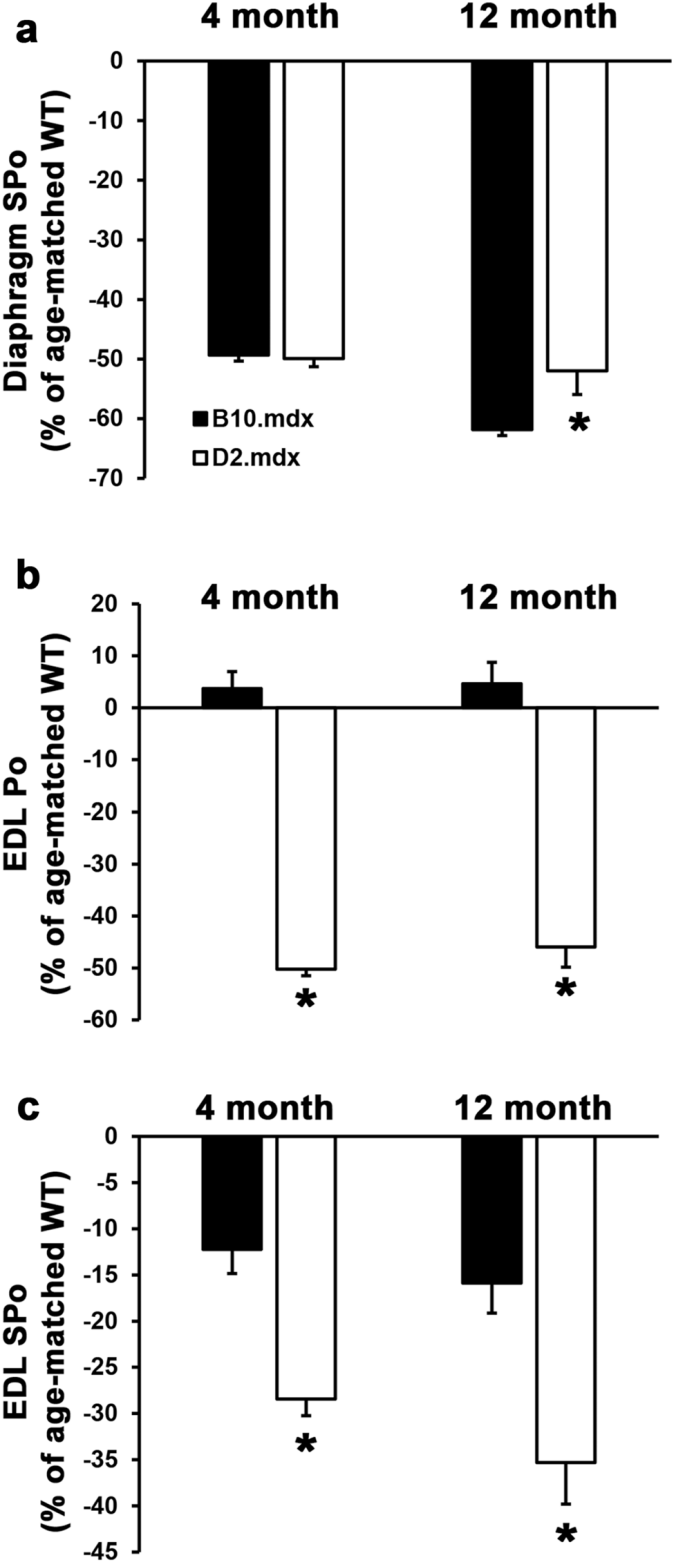
Functional comparison of diaphragm and EDL function in B10.*mdx* and D2.*mdx* mice. (**a**) Diaphragm specific tension (SPo) and (**b**) maximum force production (Po) and (**c**) SPo of the extensor digitorum longus (EDL) from 4 and 12 month-old B10.*mdx* and D2.*mdx* male mice. Values depict muscle function relative to the age- and strain-matched wild-type (WT) mice, and are presented as mean ± SEM. Statistical analysis was performed using 2-tailed Welch’s T-tests (*p < 0.05 vs. B10*.mdx* values).

Emerging interest in utilizing the D2.*mdx* mouse as a preclinical DMD model for both academic and industry purposes led us to perform a thorough characterization of the ex vivo muscle function exhibited by this line. In doing so, we performed a comparison with the traditional B10.*mdx* mouse at terminal endpoint ages commonly used for *mdx* therapeutic studies: 4, 6, 8, and 12 months of age (mo). Wild-type mice of both C57BL/10 and DBA/2J background strains were included at 4 and 12 month age groups. As shown in Table 1, wild-type mice of both strains show comparable specific tension [SPo; maximum force production (Po) normalized to muscle cross-sectional area (CSA)] of the diaphragm and extensor digitorum longus (EDL), while EDL Po values for C57BL/10 mice were significantly larger than those of DBA/2J due to larger muscle size (Table 2). Diaphragm Po values are not reported because the preparation of diaphragm strips for ex vivo functional measurements involves arbitrary excision, therefore SPo is the only parameter we can evaluate for ex vivo diaphragm force production. Force production of the diaphragm, a muscle that is severely affected in DMD, was reduced by ~ 50% in both *mdx* lines at 4 mo, compared to their respective wild-type values (Fig. 1a). During the progression to 12 mo, D2.*mdx* diaphragm SPo remained consistently at this level of deficit, whereas B10.*mdx* diaphragm SPo continued to modestly decline. Force production of the EDL demonstrated markedly different patterns between the *mdx* mouse lines, as B10.*mdx* EDL Po values actually exceed age-matched wild-type values, whereas D2.*mdx* EDL Po values are reduced by 40–50% (Fig. 1b). EDL SPo deficits for D2.*mdx* mice are also significantly larger than those of B10.*mdx* mice (Fig. 1c). This demonstrates that, unlike those of B10.*mdx* mice, the limb muscles of D2.*mdx* mice demonstrate functional decrements comparable to those of the diaphragm, thus offering a more meaningful evaluation of therapeutic efficacy of limb muscles.

**Table 1:** Ex vivo muscle function of wild-type and *mdx* mice of the C57BL/10 and DBA/2J genetic backgrounds.

Background	Genotype	Age (mo)	n	Diaphragm SPo (N/cm^2^)	EDL Po (mN)	EDL SPo (N/cm^2^)
C57BL/10	Wild-type	4	9	21.24 ± 1.30	378.37 ± 8.44	22.10 ± 1.18
		12	8	21.16 ± 0.92	454.29 ± 17.58^†^	25.10 ± 0.78
	*mdx*	4	11	10.76 ± 0.46^#^	392.32 ± 12.39	19.40 ± 0.58
		6	10	10.98 ± 0.55	459.70 ± 13.30^†^	19.52 ± 0.76
		8	22	7.09 ± 0.53^†^	420.18 ± 29.70	20.07 ± 1.33
		12	10	8.08 ± 0.37^#^	475.19 ± 18.86	21.11 ± 0.82
DBA/2J	Wild-type	4	7	19.72 ± 1.14	342.07 ± 16.68	24.43 ± 0.73
		12	7	20.57 ± 0.59	366.16 ± 12.13*	30.74 ± 0.69*^,†^
	*mdx*	4	60	9.88 ± 0.27^#^	170.26 ± 4.33*^,#^	17.48 ± 0.44^#^
		6	29	11.01 ± 0.31	216.04 ± 5.95*^,†^	22.08 ± 0.46^†^
		8	11	9.31 ± 0.65	210.64 ± 15.17*	19.33 ± 1.21
		12	13	9.87 ± 0.81^#^	197.97 ± 14.31*^,#^	19.88 ± 1.38^#^

Values are represented as mean ± SEM; Extensor digitorum longus (EDL); Maximum tetanic tension (Po); Specific Tension (SPo).

*p ≤ 0.05 vs. age- and genotype-matched C57BL/10 values.

^#^p ≤ 0.05 vs. age- and background-matched wild-type values.

^†^p ≤ 0.05 vs. strain- and genotype-matched values from previous age group.

**Table 2:** Morphological parameters of wild-type and *mdx* mice of the C57BL/10 and DBA/2J genetic backgrounds.

Background	Genotype	Age (mo)	Bwt (g)	Soleus (mg)	Gastrocnemius (mg)	Tibialis Anterior (mg)	EDL (mg)	Quadriceps (mg)
C57BL/10	Wild-type	4	28.93 ± 1.16	8.44 ± 0.23	134.96 ± 1.37	46.16 ± 0.90	10.93 ± 0.17	205.35 ± 1.71
		12	32.28 ± 0.91	11.70 ± 0.73^†^	152.09 ± 2.07^†^	59.18 ± 1.65^†^	12.33 ± 0.50	234.41 ± 5.41^†^
	*mdx*	4	33.75 ± 0.87^#^	11.96 ± 0.29^#^	177.87 ± 2.12^#^	77.62 ± 1.42^#^	12.96 ± 0.27^#^	327.49 ± 7.18^#^
		6	31.74 ± 1.04	12.38 ± 0.43	181.67 ± 10.05	74.93 ± 3.07	15.26 ± 0.66^†^	329.57 ± 13.98
		8	30.03 ± 1.13	12.16 ± 0.39	171.46 ± 6.56	74.73 ± 1.60	13.79 ± 0.41	288.48 ± 8.82^†^
		12	32.66 ± 0.46	13.66 ± 0.18^#,†^	146.55 ± 4.90^†^	67.81 ± 2.22^#,†^	14.61 ± 0.33^#^	260.59 ± 9.76^†^
DBA/2 J	Wild-type	4	26.62 ± 0.61	6.73 ± 0.17*	109.41 ± 1.72*	39.41 ± 0.71*	8.89 ± 0.22*	175.63 ± 2.68*
		12	38.67 ± 1.37*^,†^	7.95 ± 0.26*	105.93 ± 3.09*	35.70 ± 0.82*	7.55 ± 0.19*^,†^	176.98 ± 4.09*
	*mdx*	4	24.49 ± 0.54*	7.01 ± 0.36*	67.85 ± 0.79*^,#^	30.46 ± 0.50*^,#^	7.10 ± 0.24*^,#^	112.54 ± 1.41*^,#^
		6	26.36 ± 0.72*	8.38 ± 0.24*	70.45 ± 1.42*	30.16 ± 0.65*	5.78 ± 0.10*^,†^	102.89 ± 2.85*
		8	27.06 ± 0.71	8.46 ± 0.26*	63.95 ± 1.97*	30.28 ± 0.72*	7.64 ± 0.26*^,†^	98.02 ± 2.84*
		12	24.66 ± 0.74*^,#^	6.25 ± 0.19*^,#,†^	45.14 ± 2.14*^,#,†^	23.42 ± 0.85*^,#,†^	5.94 ± 0.19*^,#†^	76.27 ± 2.06*^,#,†^

Values are represented as mean ± SEM; Bodyweight (Bwt); extensor digitorum longus (EDL).

*p ≤ 0.05 vs. age- and genotype-matched C57BL/10 values.

^#^p ≤ 0.05 vs. age- and background-matched wild-type values.

^†^p ≤ 0.05 vs. strain- and genotype-matched values from previous age group.

As previously reported^4,5,10^, a major distinguishing characteristic between B10.*mdx* and D2.*mdx* is the effect of the *mdx* mutation on muscle mass (Table 2). At 4 mo, the masses of hindlimb muscles from B10.*mdx* mice are ~ 30–70% *higher* than those for wild-type C57BL/10 mice, whereas D2.*mdx* muscles exhibit ~ 20–40% lower masses than wild-type DBA/2J values. While the masses of some muscles, including the gastrocnemius (Gastroc) and quadriceps (Quad), of B10.*mdx* progressively decline towards wild-type levels at 12 mo, D2.*mdx* muscles demonstrate progressive muscle wasting to levels that are ~ 60% less than those of wild-type mice, which is more representative of the human disease. Due to the presence of branched muscle fibers in D2.*mdx* muscles^4^, which contribute to heterogeneity of muscle fiber size and ambiguity of distinct muscle fibers when evaluating muscle cross-sections, it is not currently clear if this wasting evident in D2.*mdx* mice is due to muscle fiber atrophy or hypoplasia (loss of muscle fibers). Nonetheless, these data indicate that the D2.*mdx* mouse represents a more appropriate DMD analog for investigation of muscle wasting involved with the disease.

### Histopathological features of D2.mdx skeletal muscle

**Figure 2:**
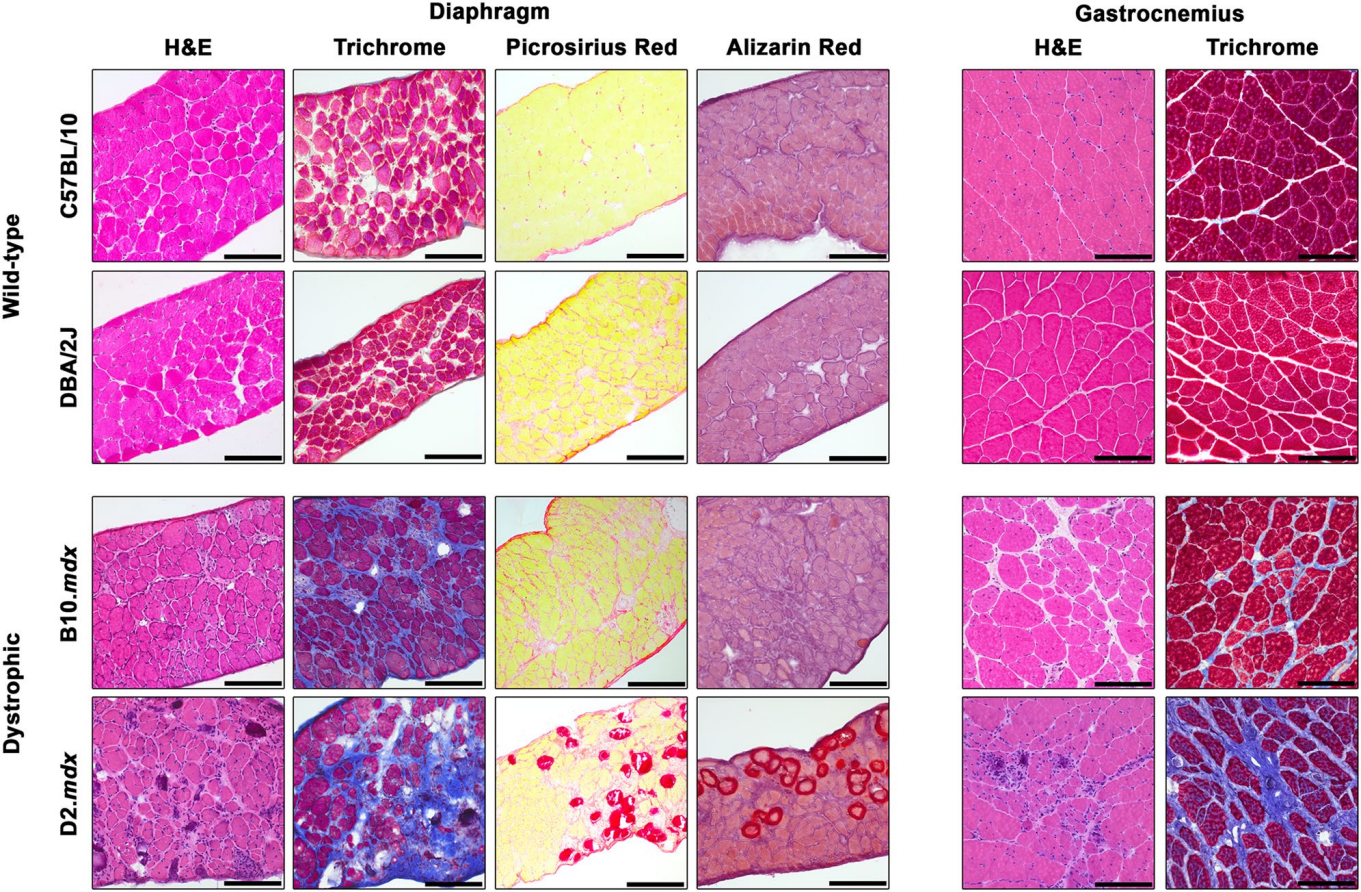
Representative histological images from 4 month-old skeletal muscle. Diaphragm and gastrocnemius sections from 4 month-old C57BL/10, DBA/2J, B10.*mdx* and D2.*mdx* are presented to display histopathological phenotypes between the mouse lines. Diaphragm muscles displayed are stained with hematoxylin and eosin (H&E), Masson’s trichrome, picrosirius red, and Alizarin red. Gastrocnemius muscles displayed are stained with H&E and Masson’s trichrome. Scale bars represent 100 µm.

**Figure 3:**
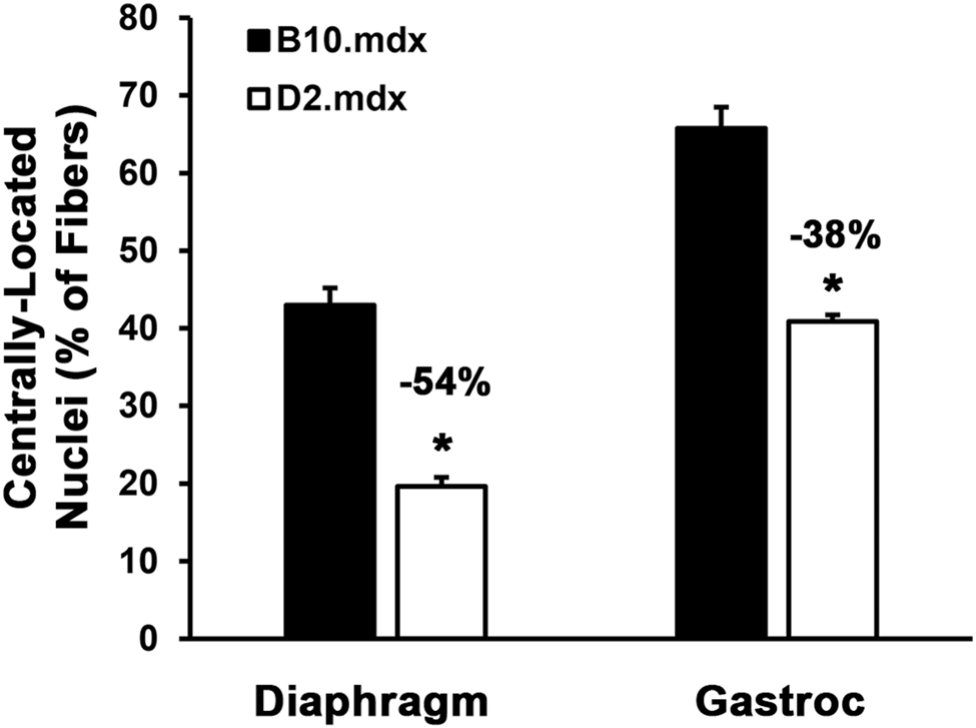
Comparison of centrally-located nuclei in B10.*mdx* and D2.*mdx* skeletal muscle. Centrally-located nuclei were quantified in the diaphragm and gastrocnemius (Gastroc) muscles of 4 mo B10.*mdx* and D2.*mdx* mice (n = 6–14). Data are presented as mean ± SEM. Statistical analysis was performed using 2-tailed Welch’s T-tests (*p < 0.05 vs. B10*.mdx* values).

Previous reports of the D2.*mdx* model suggest that this mouse line demonstrates greater histopathology, including increased skeletal muscle fibrosis, in comparison with B10.*mdx* muscles^5^. From the above-mentioned cohort, we evaluated histopathological features of the diaphragm, and gastroc, a major load-bearing muscle of the hindlimb that exhibits consistent levels of pathology, in the 4 mo age-group to see if pathological features are exacerbated in D2.*mdx* at this earlier time point. Indeed, while wild-type muscles of both genetic backgrounds show no signs of pathology, the diaphragm and gastroc of D2.*mdx* mice exhibit greater histopathology than those of B10.*mdx* mice, including greater disorganization of muscle fibers and increased fibrosis, as determined by both Masson’s trichrome (TC; stains fibrosis as blue) and picrosirius red staining (PSR; stains fibrosis as red; Fig. 2). The prevalence of muscle fibers containing centrally-located nuclei, often used as an indicator of muscle regeneration^13^, is significantly reduced in both the D2.*mdx* diaphragm and gastroc, as compared to B10.*mdx* muscles (Fig. 3), consistent with reports of reduced muscle regeneration associated with the DBA/2J genetic background^5–7^. As detailed in a previous report^14^, intramuscular calcifications are also a feature of D2.*mdx* skeletal muscle and can be visualized using Alizarin red staining (AR). In 4 mo D2.*mdx* mice, these calcification are prominent in the diaphragm and intermittently found in gastroc samples. The calcifications can also be visualized as areas of dark staining with hematoxylin & eosin (H&E) and stain the same color as fibrosis following TC and PSR staining. These data indicate that D2.*mdx* skeletal muscles do exhibit greater pathology than B10.*mdx* muscles at this earlier time point. The remainder of this report focuses on features and progression of D2.*mdx* skeletal muscle.

**Figure 4:**
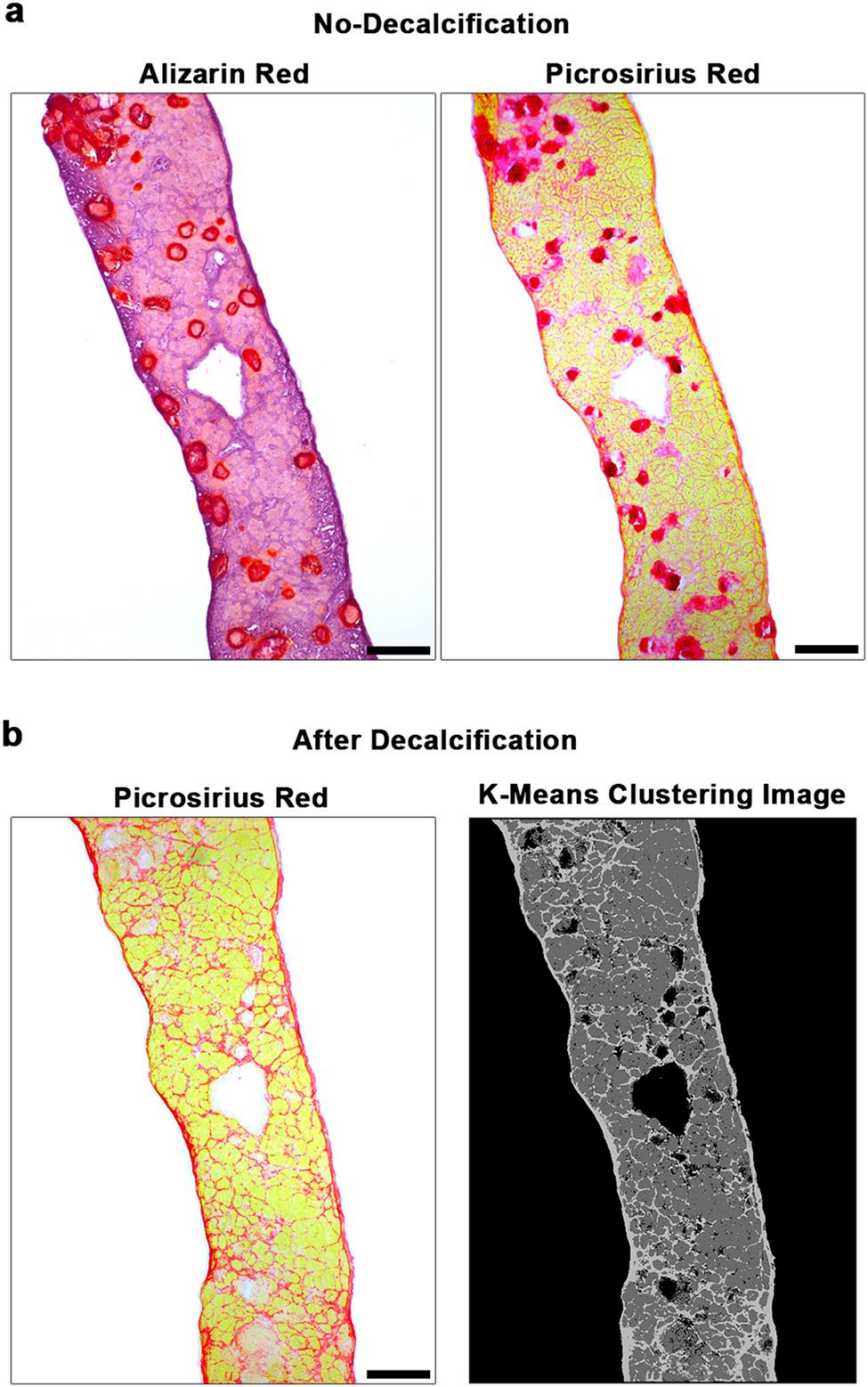
Decalcification of D2.*mdx* muscle to measure intramuscular fibrosis. Serial sections of a 4 month-old D2.*mdx* mouse diaphragm were prepared for staining with (**a**) Alizarin red and picrosirius red to demonstrate prevalence of intramuscular calcifications without decalcification treatment. (**b**) The third serial section received decalcification treatment prior to picrosirius red staining, allowing accurate histological analysis of intramuscular fibrosis using K-means clustering segmentation analysis in ImageJ software. Scale bar represents 100 µm.

Because calcifications cross-react with fibrosis-associated staining, their presence becomes a confounding factor for the quantification of fibrosis in D2.*mdx* skeletal muscle. To prevent this interference, we found that performing a decalcification step (see “Methods”) on D2.*mdx* muscle sections prior to PSR staining allows for the more accurate assessment of muscle fibrosis. As demonstrated using serial sections of D2.*mdx* diaphragm that contains extensive calcifications (Fig. 4a), decalcification of the tissue section prior to PSR staining yields a stained section that more clearly displays the fibrosis of the muscle (Fig. 4b left). Quantification of muscle fibrosis can then be performed from an image of PSR-stained muscle using the K-means clustering segmentation feature of ImageJ software (Fig. 4b right). This allows for the clear segmentation of background (white), muscle tissue (yellow), and fibrosis (red) from a PSR image, from which one can calculate the area of fibrosis as a proportion of total tissue area (muscle and fibrosis added together). If desired, the quantification of tissue calcifications can be performed using a similar methodology on non-decalcified AR stained sections or by calculating the difference between non-decalcified and decalcified PSR-stained serial sections.

**Figure 5:**
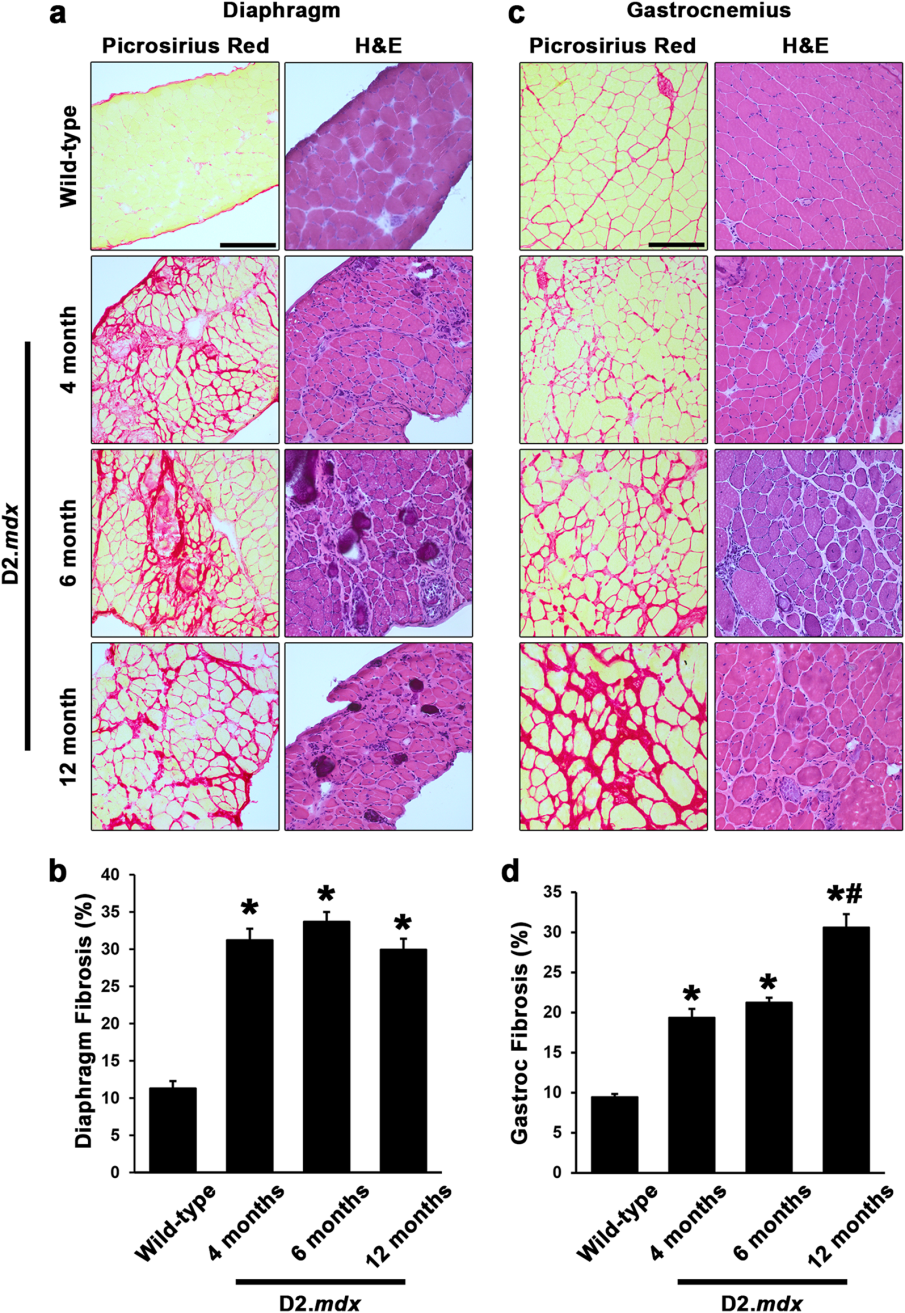
Histological quantification of D2.*mdx* skeletal muscle fibrosis. Representative picrosirius red and hematoxylin and eosin (H&E) images and fibrosis quantifications for (**a**,**b**) diaphragm and (**c**,**d**) gastrocnemius muscles from male wild-type (4 month-old DBA/2J) and 4, 6, and 12 month-old D2.*mdx* mice (n = 6–15). Values are reported as mean ± SEM, and data were analyzed using one-way ANOVA followed by Tukey post-hoc tests (*p ≤ 0.05 vs. wild-type levels; ^#^p ≤ 0.05 vs. 4- and 6-month levels).

Utilizing decalcified PSR-stained sections, we quantified muscle fibrosis in D2*.mdx* diaphragm and gastroc samples from the 4, 6, and 12 mo time points to investigate fibrotic progression in these muscles (compared to 4 mo wild-type samples). By 4 mo, the diaphragm had progressed to ~ 30% fibrosis and exhibited a plateau at this value for the remaining time points (Fig. 5a,b). The gastroc, on the contrary, exhibited progressive accumulation of muscle fibrosis, reaching levels similar to that of the diaphragm by 12 mo (Fig. 5c,d). These data indicate that therapeutic strategies to prevent the development of intramuscular fibrosis in D2.*mdx* mice should be implemented prior to the 4 mo time point in order to target fibrosis of the diaphragm, while the limb muscles offer a broader window for therapeutic evaluation.

### Insights into the time course of D2.mdx muscle pathology

**Figure 6:**
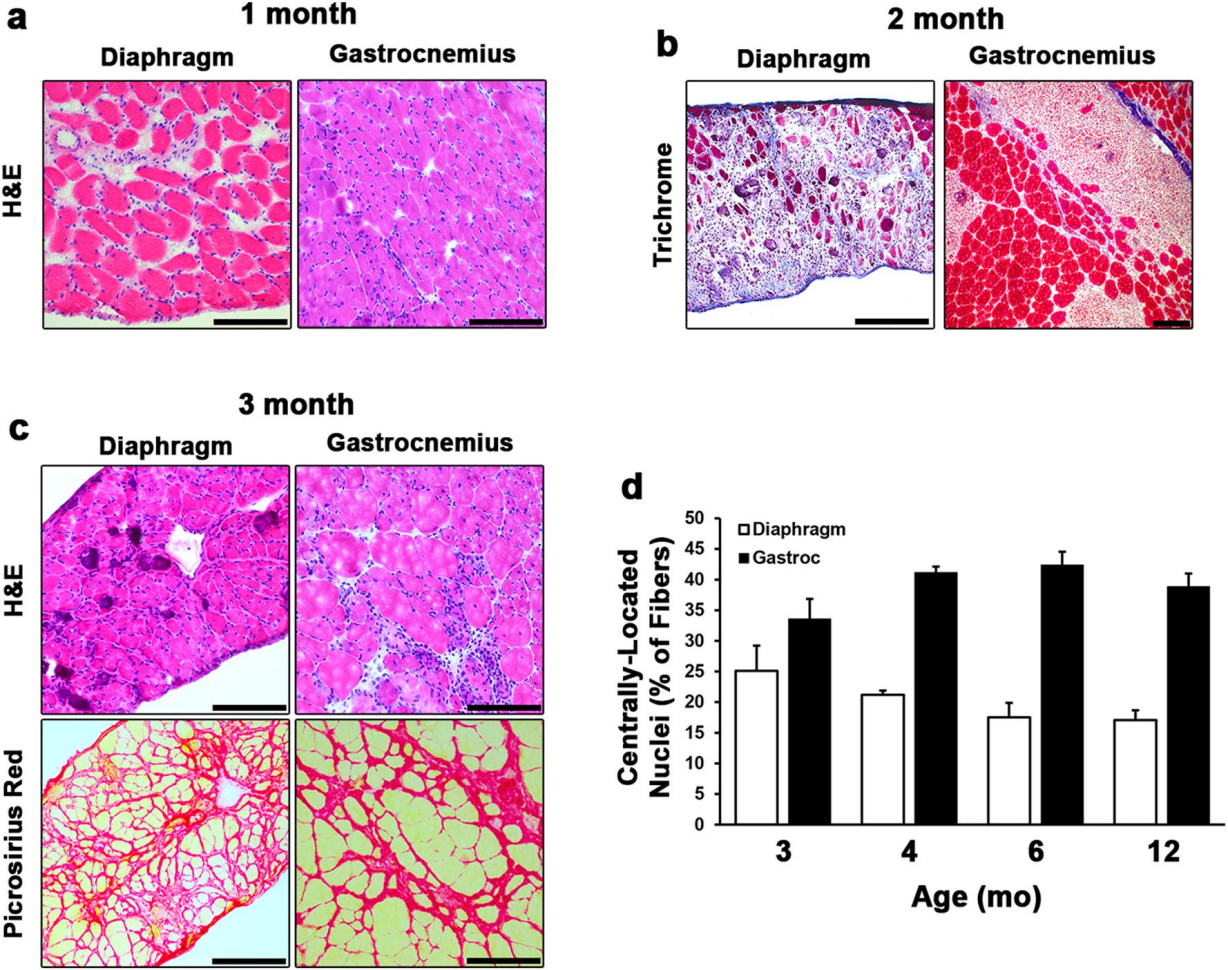
Histological time course of the development of D2.*mdx* skeletal muscle pathology. (**a**) Hematoxylin and eosin (H&E) stained sections of 1 month-old D2.*mdx* diaphragm and gastrocnemius (Gastroc) exhibiting inflammation without signs of degeneration. (**b**) Masson’s trichrome staining of 2 month-old samples reveal large degenerating lesions that precede the development of fibrosis. (**c**) H&E and picrosirius red staining of 3 month-old skeletal muscle showing major histopathological features following recovery from the degenerative stage. (**d**) Quantification of centrally-located nuclei in the diaphragms and Gastrocs of 3, 4, 6, and 12 mo D2.*mdx* mice (n = 5–12). Data were analyzed using one-way ANOVA and presented as mean ± SEM. Scale bars represent 100 µm.

The pronounced development of fibrosis and lack of degenerative lesions within the skeletal muscles evaluated in the above investigation suggest much of the muscle degeneration and inflammation associated with the D2.*mdx* model must occur prior to the 4 mo time point. We, therefore, investigated the histopathology of diaphragm and gastroc samples from 1, 2, and 3 mo D2.*mdx* mice to determine when inflammatory degeneration of skeletal muscle is evident. At 1 mo of age, neither the diaphragm or gastroc displayed signs of muscle degeneration, however, these muscles did display signs of immune infiltration and muscle fiber swelling (Fig. 6a), indicating this stage may be a pre-degenerative inflammatory stage of disease progression. At 2 mo, both muscles exhibited large lesions of degenerating muscle filled with immune infiltrate (Fig. 6b). This degenerative and inflammatory stage of muscle disease progression precedes the development of fibrosis, as indicated by TC staining. By 3 mo, the diaphragm and gastroc both contain a heterogeneous mix of mature muscle fibers, small regenerating fibers, large numbers of interstitial cells, and development of intramuscular fibrosis (Fig. 6c). These features suggest that by 3 mo, much of the muscle has regenerated from the degenerative and inflammatory stage, and fibrosis development within the muscle has begun. Interestingly, the prevalence of fibers containing centrally-located nuclei remains consistent for both the diaphragm and gastroc from 3 to 12 mo (Fig. 6d), suggesting either there is a steady rate of myonuclear addition to the fibers amongst these ages or a subset of nuclei in regenerated D2.*mdx* muscle do not become peripheral. Evidence of the latter has been reported in dystrophic muscle^15^ and following muscle injuries^16^.

**Figure 7:**
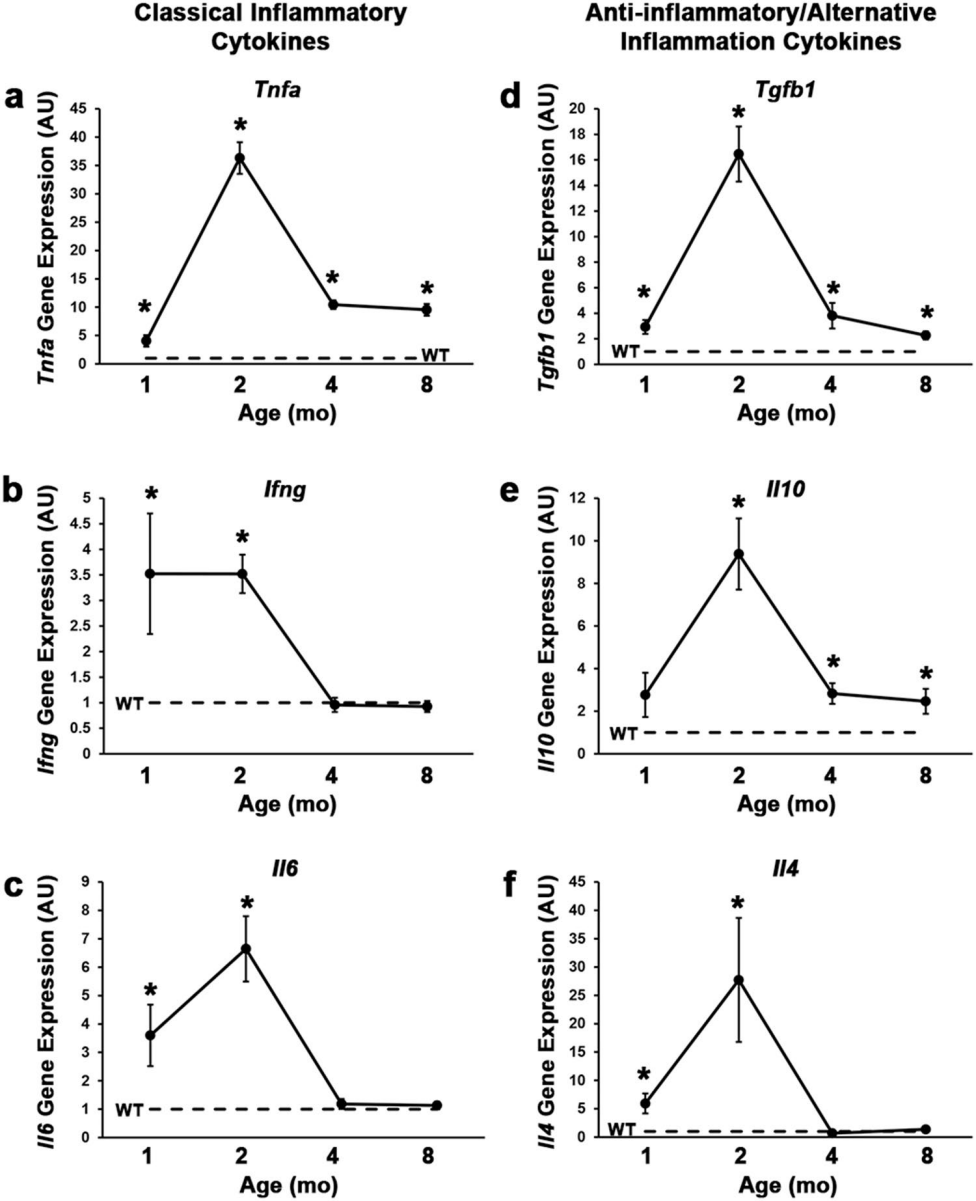
Inflammation-associated cytokine gene expression in D2.*mdx* skeletal muscle. Gene expression of (**a**) *Tnfa*, (**b**) *Infg*, (**c**) *Il6*, (**d**) *Tgfb1*, (**e**) *Il10*, and (**f**) *Il4* in the diaphragms of 1, 2, 4, and 8 month-old D2.*mdx* mice (n = 4). Values are quantified relative to those of 4 month-old DBA/2J (WT) samples (n = 4). Values are reported as mean ± SEM, and data were analyzed using one-way ANOVA followed by Tukey post-hoc tests (*p ≤ 0.05 vs. WT levels).

**Figure 8:**
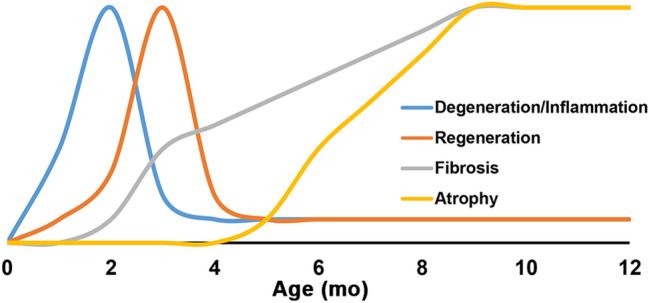
Summary of pathological features of D2.*mdx* skeletal muscle disease progression. Schematic representation of the progression of dystrophic features in D2.*mdx* skeletal muscle, including degeneration/inflammation, regeneration, fibrosis, and muscle atrophy.

In agreement with these histological observations depicting pre-degenerative inflammatory, degenerative inflammatory, and post-degenerative fibrogenic stages of D2.*mdx* skeletal muscle pathology, diaphragm gene expression profiles of cytokines associated with immune cell activity, including classical inflammatory cytokines (*Tnfa*, *Ifng*, and *Il6*; Fig. 7a–c) and anti-inflammatory/ alternative inflammation cytokines (*Tgfb1*, *Il10*, and *Il4*; Fig. 7d–f) follow similar trends of activation by 1 mo, peak expression at 2 mo, and decline/resolution by 4 mo. It is important to note that while most of these markers return to wild-type expression levels at the 4 mo time point, *Tnfa*, *Tgfb1*, and *Il10* gene expression remain elevated in the diaphragm at least through 8 mo. The occurrence of these distinct phases of D2.*mdx* skeletal muscle disease progression (summarized in Fig. 8) is an important element to consider when designing preclinical therapeutic evaluations, particularly for strategies to target inflammation and/or degeneration.

## Discussion

DMD is a devastating and lethal degenerative muscle disease, often resulting in loss of ambulation in the second decade of life and mortality by the age of 30. Because there is no cure for this disease, efforts are underway to identify potential therapeutics to slow the disease progression or transform DMD into a milder form of the disease, Becker muscular dystrophy [BMD^17,18^]. Because DMD and BMD patients both exhibit muscle weakness, degeneration, inflammation, fibrosis, and wasting, therapies that effectively target these disease features will remain a major clinical need for the foreseeable future. In this report, we provide insights into the natural history of skeletal muscle disease progression of a small animal model of DMD, the D2.*mdx* mouse, in anticipation that these data will facilitate preclinical trial design for such potential therapeutics.

While the diaphragm of B10.*mdx* has long been known to model functional losses and development of fibrosis associated with DMD^19^, the lack of overt pathology in the limb muscles of this model has complicated small animal preclinical evaluations. Herein, we show that D2.*mdx* mice demonstrate progressive muscle weakness and wasting of the limb muscles between the ages of 6 and 12 mo, whereas B10.*mdx* limb muscles are typically larger than their wild-type counterparts, also exhibiting comparable absolute forces. The increase in muscle mass and function observed in D2.*mdx* skeletal muscle between 4 and 6 months of age it likely due to maturation of muscular following recovery from the degenerative stage of muscle pathology. These observations of progressive weakness and atrophy of D2.*mdx* muscle offers a greater window for the evaluation of potential therapeutics aimed at preventing loss of muscle mass and function in dystrophic muscles, with the added benefit of comparing measured effects to wild-type values that are considerably higher than values from untreated dystrophic muscle.

In agreement with previous observations^5^, we additionally find that the histopathology, particularly fibrosis, is more severe than that exhibited by B10.*mdx* mice. The increased fibrotic phenotype of mice on the DBA/2J genetic background has been linked to a hyper-fibrotic polymorphism in LTBP4, a protein that tethers the TGFβ small latent complex to the extracellular matrix^8,20^. The DBA/2J variant of LTBP4 contains a 13 amino acid truncation within the proline-rich hinge domain, resulting in increased susceptibility of LTBP4 to proteolytic cleavage and ensuing activation of the pro-fibrotic cytokine, TGFβ^8^. Interestingly, protein alignment reports show the proline-rich hinge of human LTBP4 is considerably shorter than that of the DBA/2J mouse (41 vs 73 amino acids). This may result in activation of TGFβ in humans being more heightened than that of even DBA/2J mice, thereby contributing to the even more severe progression of the human disease. While none have been identified in this proline-rich hinge region of LTBP4, human LTBP4 single nucleotide polymorphism (SNP) haplotypes have been linked to DMD disease progression^9^, validating the translational value of LTBP4 as a modifier of disease progression in muscular dystrophies.

During the histological evaluations of this study, we found a substantial amount of intramuscular calcifications in the diaphragms of D2.*mdx* mice, which were verified using AR staining. Evidence suggests that these features, which have also been reported in DMD patient muscle biopsies^21^, express osteogenic markers, develop early in the D2.*mdx* disease progression from fibro-adipogenic progenitor cells, and dissipate at later stages of disease progression^6,14^. Their presence does, however, interfere with the histological measurement of intramuscular fibrosis using PSR and TC staining. To circumvent this issue, we have employed decalcification steps into our PSR staining protocols to eliminate these calcifications and enhance the accuracy of our PSR-mediated quantification of fibrosis. Using this strategy, we find that the natural progression of diaphragm fibrosis reaches and sustains peak levels by 4 mo, whereas the gastroc, one of the most severely affected limb muscles, exhibits progressive fibrosis at least to 12 mo. Understanding these differing time courses of intramuscular fibrosis development in the D2.*mdx* model is useful when designing preclinical investigations to prevent development of fibrosis and/or promote remodeling of intramuscular fibrosis. For example, implementation of therapeutics designed to slow or halt the progression of fibrosis will have little chance of showing efficacy in the diaphragm after 4 mo, however, may be able to demonstrate therapeutic effects in the limb muscles of D2.*mdx* mice between 3 and 12 months of age.

Consideration of the disease stages within D2.*mdx* muscle is also key to properly evaluating the efficacy of potential DMD therapeutics using this model. In general, D2.*mdx* skeletal muscle exhibits a pre-degenerative inflammatory stage at ~ 1 mo, a degenerative inflammatory stage at ~ 2 mo, and a post-degenerative fibrogenic stage that begins at ~ 3 mo, following recovery from the highly-degenerative stage. This time course differs from that of the B10.*mdx* model, where inflammatory degeneration of skeletal muscle is most prominent at ~ 1 mo^22,23^. While it is not currently clear what specifically initiates the inflammation or degeneration in the early ages of murine DMD models, the severe fibrogenic aspect of the post-degenerative stage likely arises from a combination of regenerative asynchrony associated with the degenerative stage, as early and late muscle regeneration signals co-exist within the same muscle due to unsynchronized degeneration^24^, and less robust myogenesis associated with the DBA2/J genetic background, as compared to C57-based background strains^5–7^. In agreement with this hypothesis, similar exacerbated muscle fibrosis phenotypes can also be induced in *mdx* of the C57BL/6 background by incurring repeated injuries^25^, which can promote both regenerative asynchrony and satellite cell depletion. While it remains to be determined which murine genetic background exhibits satellite cell kinetics and a muscle regeneration time course more similar to that of humans, the severe nature of DMD patient disease progression is suggestive of human myogenesis also being diminished in comparison to that of C57-based murine mouse lines.

Distinctive differences between these generalized stages of D2.*mdx* muscle pathology substantially influence the efficacious window of certain therapeutics. For example, the glucocorticoid prednisolone, a common DMD therapeutic, exhibits beneficial effects on diaphragm function when administered to D2*.mdx* mice beginning at 1 mo, whereas delaying treatment initiation to 3 mo yields no functional benefits^10^. As the primary actions of glucocorticoids are considered to be anti-inflammatory, this loss of efficacy with treatment delay is likely due to missing the highly inflammatory stage of the disease. Similarly, therapies that are designed to prevent muscle degeneration, such as gene therapy with micro-dystrophin^17^, would be best implemented prior to the degenerative stage, while those designed to affect fibrosis would be best served to have terminal endpoints at an age where intramuscular fibrosis is prominent when untreated. Utilization of these insights to design informed preclinical trials for potential DMD therapeutics may increase the efficiency of identifying efficacious treatments to advance towards clinical development.

Effective treatment options are a critical unmet need for DMD patients. The recent emergence of the D2.*mdx* mouse as a severe small animal model of DMD that better recapitulates characteristics of the human disease offers an opportunity to enhance identification of promising therapeutics for the disease. The purpose of the current report is to provide additional insights into the D2.*mdx* skeletal muscle pathology, especially features that are important from the perspective of preclinical trial design.

## Methods

### Animals

This study used male *mdx* mice of the C57BL/10 (B10.*mdx*; Jax# 001801) and DBA/2J (D2.*mdx*; Jax# 013141) backgrounds, as well as wild-type mice of the C57BL/10SnJ (Jax# 000666) and DBA/2 J (Jax# 000671), originally obtained from Jackson Laboratory. Mice were housed 3–5 mice per cage, randomly assigned into groups, provided ad libitum access to food (NIH-31 Open formulation diet; Envigo #7917), water, and enrichment, and maintained on a 12-h light/dark system. All animal procedures were approved and conducted in accordance with the University of Florida IACUC.

### Ex vivo muscle functional measurements

Maximal tetanic tension was measured in the EDL and Dp muscles by the University of Florida Physiological Assessment Core, as previously described^10^. Briefly, the muscles of anesthetized mice were dissected and placed in physiological Ringer's solution gas equilibrated with 95% O_2_/5% CO_2_. After determining optimum length, muscles were subjected to three isometric contractions (stimulated at 120 Hz for 500 ms) to determine maximum tetanic tension (Po).

### Histological evaluations

OCT-embedded muscle samples were sectioned at a thickness of 10 µm within a cryostat (Leica) maintained at – 20 °C. Hematoxylin and eosin (H&E; Polysciences, Inc.), Masson’s trichrome (TC; Polysciences, Inc.), and Alizarin Red (AR; Sigma-Aldrich) staining procedures were performed in accordance with manufacturers’ directions. Picrosirius Red (PSR) staining was performed as previously described^26^. Decalcification of muscle sections prior to PSR staining was performed by incubating slides in Formical-2000 (StatLab) for one hour at room temperature. This step is performed after the slides are fixed in paraformaldehyde and washed twice with deionized water. After completion of the Formical-2000 incubation, the decalcified slides are washed with running tap water for 3 min, rinsed in deionized water, and allowed to completely dry prior to incubation in PSR staining solution. Slides were viewed using a DMRBE microscope (Leitz/Leica) and imaged with a Leica DCF480 digital camera. Fibrosis was quantified using k-means segmentation in ImageJ software (NIH) by investigators blind to treatment groups.

### Gene expression analysis

Gene expression analysis was conducted as previously described^10^ using the following mouse-specific primers: *Tnfa* (forward) 5′-AGG CAC TCC CCC AAA AGA TG-3′ and (reverse) 5′-TTG CTA CGA CGT GGG CTA C-3′; *Il10* (forward) 5′-GCT GCC TGC TCT TAC TGA CT-3′ and (reverse) 5′-AGG CTT GGC AAC CCA AGT AA-3′; *Ifng* (forward) 5′-CGG CAC AGT CAT TGA AAG CC-3′ and (reverse) 5′-TGC ATC CTT TTT CGC CTT GC-3′; *Tgfb1* (forward) 5′-GAC TCT CCA CCT GCA AGA CCA T-3′ and (reverse) 5′-GGG ACT GGC GAG CCT TAG TT; *Il6* (forward) 5′-AAC CAC GGC CTT CCC TAC TTC-3′ and (reverse) 5′-TCT GGC TTT GTC TTT CTT GTT ATC-3′; *Il4* (forward) 5′-CCA TAT CCA CGG ATG CGA CA-3′ and (reverse) 5′-AAG CCC GAA AGA GTC TCT GC-3′; *Gapdh* (forward) 5′-AGC AGG CAT CTG AGG GCC CA-3′ and (reverse) 5′-TGT TGG GGG CCG AGT TGG GA-3′. Relative gene expression quantification was performed using the ΔΔCt method with *Gapdh* as the normalization gene.

### Statistical analysis

Statistical analysis was performed using unpaired, two-tailed Welch’s T-test and one-way ANOVA followed by Tukey HSD post-hoc tests (α = 0.05), where appropriate. Values are displayed as mean ± SEM, unless otherwise indicated.

## Data Availability

The datasets generated during and/or analyzed during the current study are available from the corresponding author on reasonable request.
